# In-hospital outcome of patients with culture-confirmed tuberculous pleurisy: clinical impact of pulmonary involvement

**DOI:** 10.1186/1471-2334-11-46

**Published:** 2011-02-21

**Authors:** Chin-Chung Shu, Jann-Tay Wang, Jann-Yuan Wang, Li-Na Lee, Chong-Jen Yu

**Affiliations:** 1 Department of Traumatology, National Taiwan University Hospital, Taipei city, Taiwan; 2 Department of Internal Medicine, National Taiwan University Hospital, Taipei city, Taiwan; 3 Department of Laboratory Medicine, National Taiwan University Hospital, Taipei city, Taiwan

## Abstract

**Background:**

Outcomes for hospitalized patients with tuberculous pleurisy (TP) have rarely been reported, and whether or not pulmonary involvement affects outcomes is uncertain. This study aimed to analyze the in-hospital mortality rate of culture-confirmed TP with an emphasis on the clinical impact of pulmonary involvement.

**Methods:**

Patients who were hospitalized for pleural effusion (PE) of unconfirmed diagnosis and finally diagnosed as TP were identified. We classified them according to the disease extent: isolated pleurisy (isolated pleurisy group) and pleurisy with pulmonary involvement (pleuro-pulmonary group).

**Results:**

Among the 205 patients hospitalized before the diagnosis was established, 51 (24.9%) belonged to the isolated pleurisy group. Compared to the pleuro-pulmonary group, patients in the isolated pleurisy group were younger, had fewer underlying co-morbidities, and presented more frequently with fever and chest pain. Fewer patients in the isolated pleurisy group had hypoalbuminemia (< 3.5 g/dL) and anemia. The two groups were similar with regards to PE analysis, resistance pattern, and timing of anti-tuberculous treatment. Patients who had a typical pathology of TP on pleural biopsy received anti-tuberculous treatment earlier than those who did not, and were all alive at discharge. The isolated pleurisy group had a lower in-hospital mortality rate, a shorter length of hospital stay and better short-term survival. In addition, the presence of underlying comorbidities and not receiving anti-tuberculous treatment were associated with a higher in-hospital mortality rate.

**Conclusion:**

In culture-confirmed tuberculous pleurisy, those with pulmonary involvement were associated with a higher in-hospital mortality rate. A typical pathology for TP on pleura biopsy was associated with a better outcome.

## Background

Tuberculosis (TB) remains a global health problem even though it has nearly been eradicated in some developed countries [1,2]. The incidence in 2005 was 76 per 100,000 persons in Taiwan, 80 per 100,000 in the Republic of Korea, and 600 per 100000 in South Africa [3,4]. TB remains a leading cause of mortality in many countries. The mortality rate has been reported to be 6% in those with pulmonary TB, and as high as 31% in those with disseminated TB [3,4].

Because of variable manifestations and the difficulty in collecting clinical samples, extra-pulmonary TB is usually difficult to diagnose early [5]. Tuberculous pleurisy (TP) is the second most common extra-pulmonary infection [5], and accounts for approximately 5% of all forms of TB [6]. The gold standard for the diagnosis of TP is still mycobacterial culture of pleural effusion (PE), pleura tissue and respiratory specimens, which requires weeks to yield. The treatment could thus be delayed, resulting in an increased mortality rate [7]. For those requiring hospitalization, the mortality rate is further increasing due to increasingly severe infections and weaker host states [8,9].

The prognostic factors for hospitalized patients with TP are unclear. Only limited information is available on whether or not pulmonary involvement has a negative prognostic impact [6,10]. However, the mortality rate is high in tuberculosis patients if they are not promptly diagnosed and treated [11]. Therefore, we conducted this retrospective study to investigate the in-hospital mortality rate of culture-confirmed TP with an emphasis on the clinical impact of pulmonary involvement.

## Methods

### Subjects of study

This retrospective study was conducted in a tertiary-care referral center in northern Taiwan by reviewing the medical charts as in our previous study [12]. The study was approved by the Institutional Review Board of the Research Ethics Committee of National Taiwan University Hospital (No.: 200809076R). The informed consent was deemed unnecessary for this retrospective study. We reviewed the mycobacterial laboratory registry database of the hospital and identified all patients with PE specimens sent for mycobacterial culture from January 2001 to December 2008. Among them, those who were hospitalized for PE before the diagnosis of TP was established by mycobacterial culture for PE were included for further investigation. Patients were classified into two groups according to the disease extent of TB: the isolated pleurisy group and pleuro-pulmonary group. The former was considered if all respiratory samples from a patient were culture-negative for *M tuberculosis *and there were no pulmonary parenchymal lesions compatible with active TB on chest radiographs, defined as new patch(es) of consolidation, collapse, lymphadenopathy, mass or nodule, cavitary lesion or infiltrate without other proven etiology [13]. The others were classified into the pleuro-pulmonary group.

### Data collection

Patient data were collected by reviewing medical records and recorded in a standardized case report form by one chest physician, then verified by another physician from July 2009 to December 2009. Data included age, gender, underlying co-morbidities, initial symptoms, laboratory data and radiographic findings when the index PE sample was collected, as well as the course and outcome of anti-tuberculous treatment. Mycobacterial culture and susceptibility testing were performed according to standard procedures [3,14]. In our hospital, acid-fast smear and mycobacterial culture for pleural effusion samples were routinely performed in cases of lymphocytic pleural exudate by Light's criteria [15]. For patients with adequate cough power, sputum samples were collected by spontaneous expectoration after explanation without supervision. For the others, sputum samples were collected by a nurse using a suction tube inserted through mouth or nasal cavity. We routinely ordered at least three sets of mycobacterial cultures for sputum samples collected from each patient. Bilateral lesions were considered if the contra-lateral lung or pleural cavity were involved. Three histological findings of pleura tissue were considered typical for TP: (1) granulomatous inflammation, (2) caseous necrosis, and (3) the presence of acid fast bacilli [16].

Patients received standard short-course anti-TB treatment with isoniazid (INH), rifampicin (RIF), ethambutol (EMB) and pyrazinamide (PZA) for the initial 2 months, and INH plus RIF for the following 4 months. The standard regimen was modified if drug resistance or adverse effects were encountered [17,18]. Patients were followed for at least 6 months after the index PE samples were collected, or until death or loss of follow-up. Residual pleural thickening (RPT) on radiographs after 6 months of treatment was defined as minor if the pleural thickness was less than 10 mm, or major if equal to or greater than 10 mm. One pulmonologist and one radiologist, both blinded to the clinical data, interpreted the chest radiographs. If their opinions differed, the films were further reviewed by another senior pulmonologist blinded to the results.

### Statistics

The inter-group differences were compared by using the independent *t *test for numerical variables and the *chi*-square test or Fisher's exact test for categorical variables as appropriate. Survival curves were generated using the Kaplan-Meier method and were compared using the log-rank test. Variables having a significant difference (*p *< 0.05) for in-hospital mortality in univariate analysis were further tested by logistic regression with the forward conditional method.

## Results

During the 8-year study period, a total of 496 samples from 412 patients out of 24,759 PE samples yielded *M. tuberculosis*. Among them, 205 patients were hospitalized when TP was culture-confirmed. The indications for hospitalization were intolerant fever or dyspnea in 99, massive and/or loculated PE in 51, prolonged symptoms (> 14 days) in 51, and presence of lung mass in 14. Among the 205 patients, 51 were further classified into the isolated pleurisy group. The other 154, including 97 (63%) whose sputum samples were culture-positive for *M. tuberculosis*, were classified into the pleuro-pulmonary group. A total of 3,112 patients had culture-confirmed pulmonary TB.

The clinical characteristics of the patients with TP are listed in Table 1. Patients in the isolated pleurisy group were younger and less frequently had underlying co-morbid illnesses than those in the pleuro-pulmonary group. Among patients aged less than 65 years, underlying co-morbid illnesses were still less common in the isolated pleurisy group (11% *vs*. 45%, *p *= 0.003), but similar between the two groups in those aged 65 years or older (48% *vs*. 47%, *p *= 0.968). Malignancy and diabetes mellitus were the most common co-morbidities in the two groups. The serostatus of *Human Immunodeficiency Virus *(HIV) was tested in 63 (31%) patients and was positive in 6, with no inter-group difference. Of the 142 patients with unknown HIV serostatus, all were free of other acquired immunodeficiency syndrome (AIDS)-defined illness during follow-up. Male predominance was noted in both groups. The duration of symptoms was about 17 days, and 51% of the patients in the isolated TP group presented with fever. Fever was also more common in those aged less than 65 years (54% *vs*. 23%, *p *< 0.001), without underlying co-morbidities (40% *vs*. 24%, p = 0.017), or without hypoalbuminemia (defined as a serum level of albumin less than 3.5 g/dL) (42% *vs*. 26%, *p *= 0.049). More patients in the isolated pleurisy group suffered from chest pain, but dyspnea was most common in the pleuro-pulmonary group.

**Table 1:** Clinical characteristics of the patients with tuberculous pleurisy

	Isolated pleurisy group (N = 51)	Pleuro-pulmonary group (N = 154)	*p *value
Age (years)	52.6 [27.7]	70.4 [16.5]	< 0.001
Age ≥ 65 years	23 (43%)	114 (74%)	< 0.001
Male gender	35 (69%)	114 (74%)	0.453
Underlying co-morbid condition*	14 (27%)	72 (47%)	0.015
Diabetes mellitus	4 (8%)	27 (18%)	0.118
Malignancy	7 (14%)	22 (14%)	0.921
Renal failure	3 (6%)	16 (10%)	0.575
Cirrhosis of liver	2 (4%)	7 (5%)	1.000
Autoimmune diseases	0	7 (5%)	0.198
Acquired immunodeficiency syndrome^#^	1 (2%)	5 (3%)	1.000
Symptoms			
Duration, days	17.8 [31.0]	17.3 [27.4]	0.923
Cough	2 (4%)	19 (12%)	0.112
Fever	26 (51%)	43 (28%)	0.002
Dyspnea	13 (25%)	51 (33%)	0.360
Chest pain	6 (12%)	3 (2%)	0.007
Others†	4 (8%)	38 (25%)	0.010

Data are no. (%) or mean [SD]

* Three and twelve in the isolated pleurisy group and pleuro-pulmonary group, respectively, had two underlying co-morbid conditions.

† Other symptoms included gastrointestinal symptoms, consciousness change and other non-specific symptoms.

^# ^63 patients received human immunodeficiency tests.

The results of laboratory tests revealed that more patients in the pleuro-pulmonary group had anemia and hypoalbuminemia (Table 2). The two findings were also significantly associated with an age of 65 years or older (*p *= 0.008 and *p *< 0.001, respectively) and underlying comorbid condition (*p *< 0.001 for both). Pleural biopsy was performed in 69% (n = 35) of the isolated pleurisy group and in 33% (n = 51) of the pleuro-pulmonary group, with 75.6% (n = 65) showing granulomatous inflammation with/without caseating changes. Patients with a typical pleural pathology were treated earlier after index PE culture than those who did not have a typical pleural pathology (8.0 *vs*. 14.6 days, *p *< 0.001). The resistance patterns were similar between the isolated pleurisy group and pleuro-pulmonary group. Nineteen patients had resistance against at least one first-line drug, and four patients had multidrug-resistant TB. Radiographically, the isolated pleurisy group had fewer patients with bilateral lesions and more with loculated PE.

**Table 2:** Laboratory and radiographic findings of the patients with tuberculous pleurisy

	Isolated pleurisy group (N = 51)	Pleuro-pulmonary group(N = 154)	*p *value
Positive AFB in PE	1 (2%)	4 (8%)	1.000
Receiving pleura biopsy	35 (69%)	51 (33%)	< 0.001
Granulomatous inflammation	25 (71%)	40 (78%)	0.458
Pretreatment resistance pattern			
Anyone-drug resistance	5 (10%)	14 (9%)	0.837
Isoniazid	4 (8%)	13 (8%)	0.931
Rifampicin	1 (2%)	3 (2%)	0.977
Ethambutol	2 (4%)	4 (3%)	0.605
Multidrug resistance	1 (2%)	3 (2%)	0.977
Radiographic findings			
Bilateral lesions	5 (10%)	61 (40%)	< 0.001
Loculated PE	22 (43%)	23 (15%)	< 0.001
PE analysis			
Leukocyte (/μL)	3016 [5297]	1938 [4649]	0.239
Lymphocyte (%)	82 [24]	75 [25]	0.092
Neutrophil (%)	11 [18]	17 [21]	0.096
Total protein (g/dL)	4.6 [1.0]	4.2 [3.1]	0.264
Lactate dehydrogenase (U/L)	965 [593]	1166 [1837]	0.292
Glucose (mg/dL)	87 [42]	104 [62]	0.202
Blood tests			
Leukocyte > 11000 or < 4000/μL	6 (12%)	29 (19%)	0.286
Anemia	21 (41%)	98 (60%)	0.003
Albumin < 3.5 g/dL	19 (37%)	83 (54%)	0.007
Total bilirubin > 1.2 mg/dL	5 (10%)	20 (13%)	0.800

AFB = acid-fast bacilli, PE = pleural effusion

Data are no. (%) or mean [SD]

* Hemoglobin < 12 g/dL in men or < 11 g/dL in women was considered anemia.

A total of 29 patients did not receive anti-tuberculous treatment (Table 3). Of them, 19 patients in the pleuro-pulmonary group died before the diagnosis of TP was culture-confirmed. Another five in the pleuro-pulmonary group and five in the isolated pleurisy group were discharged and lost to follow-up before the results of mycobacterial culture became available. Among those who received anti-tuberculous treatment, the median interval from the sampling date of index PE specimen to anti-tuberculous treatment was 6 days in the isolated pleurisy group and 9 days in the pleuro-pulmonary group (*p *= 0.367) (Table 3). About two-thirds of each group received anti-tuberculous treatment within 2 weeks after the index PE samples were collected. Nine patients underwent video-assisted thoracoscopy for decortication and 19 received tube thoracostomy. There was no significant between-group difference.

**Table 3:** Treatment and outcomes

	Isolated pleurisy group (N = 51)	Pleuro-pulmonary group (N = 154)	*p *value
Anti-tuberculous treatment	46 (90%)	130 (84%)	0.305
Tube thoracostomy or decortication	4 (8%)	24 (16%)	0.163
Days-to-treatment	6 [26.8]	9 [15.6]	0.367
Within 2 weeks	35 (69%)	86 (56%)	0.264
More than 2 weeks	11 (21%)	44 (28%)	
Not treated	5 (10%)	24 (16%)	
Residual pleura thickening*			
≥ 10 mm	10 (29%)	24 (35%)	0.542
< 10 mm	25 (71%)	45 (65%)	
In-hospital mortality rate	2 (4%)	37 (24%)	0.001
Length of hospital stay: days	22 [20.8]	33 [27.9]	0.003

Data are no. (%) or mean [SD]

* After six months of anti-tuberculous treatment, only 36 patients in the isolated pleurisy group and 72 in the pleuro-pulmonary group were still being followed in our hospital.

**Figure 1:**
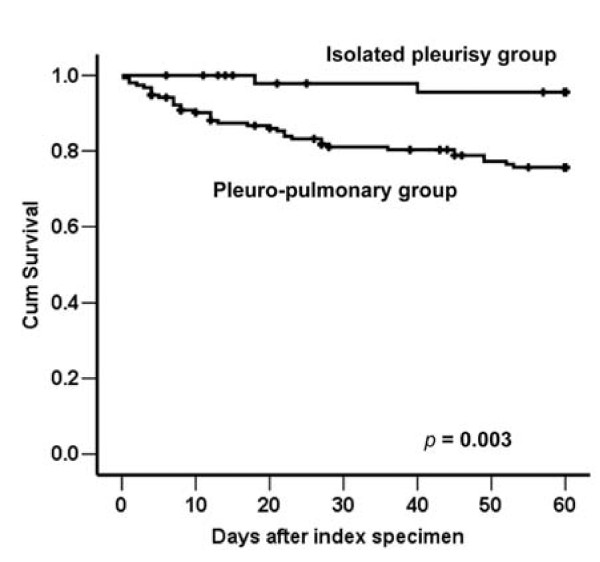
**Survival curves were plotted using the Kaplan-Meier method for patients with tuberculous pleurisy according to the disease extent (the isolated pleurisy group and pleuro-pulmonary group)**. Black dots represent patients who were still alive at the end of the study.

Outcome analysis showed that the pleuro-pulmonary group had a higher in-hospital mortality rate and longer length of hospital stay than the isolated pleurisy group (Table 3). Among the 39 patients who died before discharge, 2 patients belonged to the isolated pleurisy group and both had underlying malignancy. The remaining 37 patients had pleuro-pulmonary TB. Among them, 24 (65%) of them had underlying diseases, including malignancy in 12, diabetes mellitus in 6, end-stage renal disease in 6, liver cirrhosis in 4, and autoimmune disease requiring immunosuppressant in 1 (5 of them had two underlying diseases). None of the 39 patients had HIV infection. The cause of death was multi-organ failure in 28, refractory respiratory failure in 10, and massive gastrointestinal bleeding in 1. Among those who died of multi-organ failure, only three were documented to have concomitant bacteremia or fungemia. The role of pleuro-pulmonary involvement continued in 2-month survival analysis (Figure 1, *p *= 0.003). Within the first 6 months of treatment, 67 patients died and 30 were lost to follow-up. Of the remaining 108 patients, 35 of the 36 patients in the isolated pleurisy group and 69 of the 72 in the pleuro-pulmonary group had received chest radiography after six months. The proportion of patients with RPT ≥ 10 mm was similar in the two groups (*p *= 0.542).

The 65 patients with typical pleura pathology for TP were all alive at the time of discharge, whereas only 101 patients (72%) of the remaining 140 patients were alive at discharge (*p *< 0.001 by the *chi*-square test). Thus, we concluded that "typical pleura pathology" was a significant predictor of in-hospital mortality, and then excluded the 65 patients from multivariate logistic regression analysis. The results showed that pulmonary involvement, underlying comorbidity and not receiving anti-TB treatment were independent risk factors of in-hospital mortality (Table 4).

**Table 4:** Factors possibly associated with in-hospital mortality

Characteristics		Unlvariate *p *value	Multivariate *p *value	Multivariate OR (95% CI.)
Age	≥ 65 years	0.025	0.865	
	< 65 years			
Underlying co-morbid illness	Yes	0.001	0.036	2.60 (1.06~6.38)
	No			
Disease extent	Pleuro-pulmonary	0.001	0.014	8.67 (1.56~48.27)
	Isolated pleurisy			
Serum albumin level	< 3.5 g/dL	< 0.001	0.344	
	≥ 3.5 g/dL			
Anemia	Presence	0.008	0.444	
	Absence			
Drug resistance	Anyone	0.370		
	All sensitive			
Radiographic finding	Bilateral	0.002	0.211	
	Unilateral			
Days to anti-tuberculous treatment	No treated	< 0.001	< 0.001	12.17 (3.88~38.14)
	> 14 days	0.164	0.914	1.06 (0.38~2.92)
	≤ 14 days			

The 65 patients with typical pleural pathology for TP were all alive at discharge, whereas 39 of the remaining 140 patients died in hospital (*p *< 0.001 by the *chi*-square test). Therefore, logistic regression was performed on the 140 patients who had not received a pleural biopsy or had no typical pleural pathology for TP.

## Discussion

The pleural cavity is a common site of involvement in extra-pulmonary TB [5,16]; however, the outcomes and prognostic factors are unclear in hospitalized populations. In this retrospective study, those with pleuro-pulmonary TP accounted for three-fourths of all TP patients requiring hospitalization and had a higher in-hospital mortality rate. The in-hospital mortality rate was also higher among patients who had underlying comorbidities, did not receive anti-TB treatment and had no typical pleural pathology for TP.

Although the residual RPT was similar, our analysis showed that the in-hospital mortality rate was six-fold higher in patients with pulmonary involvement than those with isolated pleurisy (24% *vs*. 4%). Compatible with a previous report showing high mortality in hospitalized TB patients [8], our previous study revealed that patients with neutrophil-predominant TP had an in-hospital mortality rate of 36% [7]. There are several possible explanations for the high in-hospital mortality rate of patients with TP, especially for those with pulmonary involvement. Because patients with isolated pleurisy are more likely to have local and systemic inflammatory symptoms such as chest pain and fever rather than hypoalbuminemia, pulmonary involvement probably represents an extensive and serious infection in a compromised and malnourished host. Another possible explanation is that TB is usually at the top of the list of the differential diagnoses for lymphocyte-rich pleurisy [15], whereas it accounts for only 1~2% of the etiologies for pneumonia [19], thus treatment is frequently delayed. Although a delay in treatment for more than 14 days was not an independent poor prognostic factor, the 19 cases of rapid mortality in our study suggest that TP could be an immediately fatal disease, and timely and effective anti-tuberculous treatment is vital, especially for those with pleuro-pulmonary involvement.

However, two previous studies failed to demonstrate a difference in clinical outcomes between isolated TP and pleuro-pulmonary TB [6,10]. Again there are several possible explanations. First, the previous studies analyzed survival after completing anti-TB treatment and relapse, rather than in-hospital mortality. These long-term outcomes were more likely to be confounded by other factors, such as age, underlying co-morbidity, and socioeconomic status. Second, those needing admission were probably more severe cases, especially in a referral medical center. Finally, the patients in the previous reports were younger, around the fifth to early sixth decade, and less than 10% of them had underlying comorbid conditions [16,20].

Our results revealed that histologic examination of the pleural biopsy is the key step for the early diagnosis of TP, because it can effectively demonstrate a typical pathology of TP in more than three-fourths of patients within 3 days, which is higher than the yield rate of mycobacterial cultures for PE samples (11%) [21]. Moreover, even when using the fluorometric BACTEC technique, the results of mycobacterial culture still take one to two weeks [22]. Hence, a typical pleura pathology could result in the early diagnose of TP and improved outcomes. Therefore, for in-patients with lymphocyte-rich PE, the possibility of tuberculosis should be kept in mind and pleural histology should be performed at an early stage if clinically feasible. For the early diagnosis of TP, biomarkers in pleural effusion such as adenosine deaminase and interferon-gamma have been shown to be helpful, but further investigations are needed for the application of nucleic acid amplification tests and interferon-gamma release assays [23,24].

Our study has several limitations. First, in this retrospective study, the number of patients with culture-confirmed TP could have been underestimated because mycobacterial cultures were not routinely performed for every PE sample, and most studies show the sensitivity to be less than 30% [16]. Therefore, the patients with culture-negative TP might have been missed. However, the selected patients were all true cases of TP and represented a homogenous population for detailed analysis. Second, the 6-month follow-up rate was less than 90%. Third, our study population was selected from a large medical referral center. Whether our findings can be extrapolated to all TP patients should be further confirmed.

## Conclusion

Our study revealed that for hospitalized patients with TP, pulmonary involvement, underlying comorbidities, no typical pleura pathology and not receiving anti-TB treatment were associated with a worse in-hospital outcome. Aggressive examination, such as pleural biopsy, for pleural effusion with unknown cause is suggested for the early diagnosis and treatment if clinically appropriate.

## Competing interests

All of the authors declare no competing interest of any nature or kind in related products, services, and/or companies.

## Authors' contributions

JYW, JTW, and CCS designed the study, collected all relevant data and wrote the manuscript. CJY, and LL contributed to analyzing data. All authors read and approved the final manuscript.

## Pre-publication history

The pre-publication history for this paper can be accessed here:

http://www.biomedcentral.com/1471-2334/11/46/prepub
